# Logarithmic Strain Model for Nonlinear Load Cell

**DOI:** 10.3390/s19163486

**Published:** 2019-08-09

**Authors:** Young-Dae Hong, Bumjoo Lee

**Affiliations:** 1 Department of Electrical and Computer Engineering, Ajou University, Suwon 16499, Korea; 2 Department of Electrical Engineering, Myongji University, Yongin 17058, Korea

**Keywords:** nonlinear load cell, logarithmic strain model, force measurement

## Abstract

General load cells have typically constant sensitivity throughout the measurement range, which is acceptable for common force measurement systems. However, it is not adequate for high-performance control and high-stroke applications such as robotic systems. It is required to have a higher sensitivity in a small force range than that in a large force range. In contrast, for large loading force, it is more important to increase the measurement range than the sensitivity. To cope with these characteristics, the strain curve versus the force measurement should be derived as a logarithmic graph. To implement this nonlinear nature, the proposed load cell is composed of two mechanical components: an activator, which has a curved surface profile to translocate the contact point, and a linear torque measurement unit with a moment lever to measure the loading force. To approximate the logarithmic deformation, the curvature of the activator was designed by an exponential function. Subsequent design parameters were optimized by an evolutionary computation.

## 1. Introduction

A load cell is a transducer that quantitatively converts mechanical force and/or torque to an electrical signal [1,2,3]. When the load is applied to the sensor system, the elastic part of the mechanical structure is deformed on a micro scale. Subsequently, mechanical strain is converted to voltage by the strain gauge, which has a resistance proportional to its strain. Finally, this voltage reading is translated into digital information by ADC circuits. Load cells are employed in many applications to quantitatively measure the applied force in a system [4,5,6]. Especially in robotics, they enable high-precision force control by providing force information such as actuator output torque and contact reaction force [7,8,9]. For applications that have a high-force contact with the environment, the load cell needs to measure large forces [10,11,12]. When the measurement range increases, however, it is difficult to acquire force information with sufficient sensitivity in the small-force range because conventional load cells operate on a linear scale [13,14,15]. This is obvious and inevitable owing to the limitations of the ADC resolution. Therefore, to use this resource, or resolution, effectively, it should be properly assigned according to the input range. Generally, a biological system is able to interpret small differences for a low stimulus but not distinguish an identical difference for a high stimulus. This illustrates that the system has high sensitivity at a low input value and low sensitivity at a high input value. Similarly, the load cell has to follow a nonlinear behavior to cover the wide measurement range with sufficient performance [1]. In other words, at a low value of applied force, it requires high sensitivity and vice versa. To figure out these characteristics, a mathematical model was formulated and subsequent design parameters were determined.

## 2. Logarithmic Strain Model

### 2.1. Logarithmic Map of Sensory System

In sensory system, output signal is generated by input stimulus. Thus, by measuring the output signal, input stimulus can be estimated indirectly. The higher the precision and the wider the operating range, the better the performance. Since there is a trade-off between the precision and the operating range, an optimal mapping model is required. In this section, mathematical model of input and output for the general sensory system is reviewed [1]:

The sensitivity of a sensory system is expressed as the derivative of output signal with respect to input signal:

(1) $$\rho =\frac{dy}{dx}$$

where x and y represent applied input stimulus and consequent sensory output, respectively. The conventional linear sensory system adapts a linear model to generate the output signal proportional to the input stimulus. Therefore, the sensitivity of the sensor is constant over the entire operating range. For the efficiency aspect, however, it is more reasonable that the sensitivity in a small input should be larger than the sensitivity in a large input and vice versa. To capture this property, a new nonlinear property was proposed in which the sensitivity is inversely proportional to the applied force as follows:

(2) $$\rho =\alpha \frac{1}{x+\beta^{*}}$$

where α(>0) and $\beta^{*}\left(>0\right)$ denote a proportional coefficient and offset constant to limit the initial sensitivity as a finite value at the origin, respectively. The differential relationship between the input and the output is now derived by adding (1) and (2):

(3) $$dy=\alpha \frac{dx}{x+\beta^{*}}$$

Integrating (3) gives the following logarithmic function:

(4) $$y=\alpha {log}_{e}\left|x+\beta^{*}\right|+c$$

where c denotes the integral constant. Output should not arise unless the input is applied. This initial condition is applied to (4) and $\beta^{*}$ is substituted with a new parameter ($\beta =1/\beta^{*}$) to derive the map from the applied input to the sensory output:

(5) $$y=\alpha {log}_{e}\left|1+\beta x\right|$$

From (5), it can be known that the ideal mapping from input stimulus to the output signal is described by logarithmic function.

### 2.2. Mechanism Design

**Figure 1:**
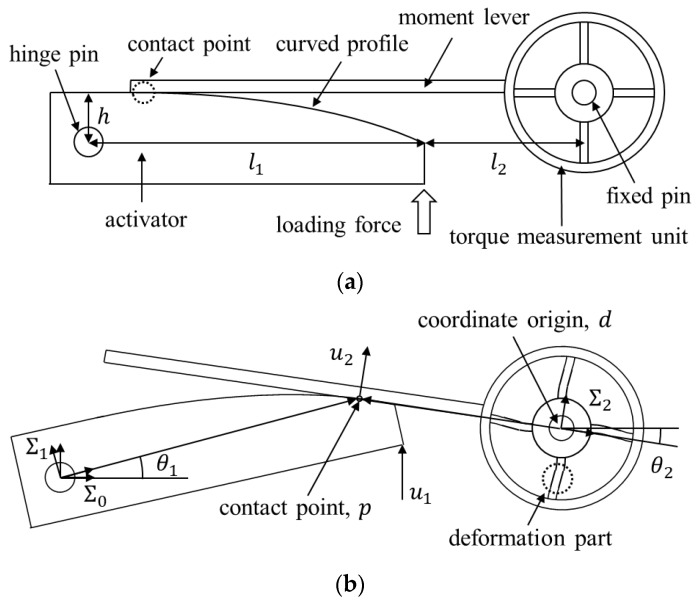
Proposed nonlinear load cell. (**a**) Initial configuration of the mechanical parts. (**b**) Deformation configuration of the activator and the measurement unit according to the loading force.

To implement the logarithmic map in load cell, novel mechanism is proposed, which has two mechanical components: an activator and a torque measurement unit. The activator transfers the loading force to the moment lever with a small rotational displacement around the hinge pin. This causes the deformation part to generate strain to keep the moment balance. As the contact point is translocated according to the linear loading force, the torque for the measurement unit increases nonlinearly. Figure 1 illustrates the free body diagram of the proposed load cell with a loading force. When the force is loaded, the activator is rotated by $\theta_{1}$ and the consequent deformation of the torque measurement unit is demonstrated by $\theta_{2}$, as shown in the figure. The coordinate system was attached on each frame, i.e., the activator, $\Sigma_{1}$, the measurement unit, $\Sigma_{2}$ and the ground coordinate system, $\Sigma_{0}$. The contact point and the coordinate origin of the measurement unit are denoted by $p={\left[x_{c}\;\;\;y_{c}\right]}^{T}$ and $d={\left[d_{x}\;\;\;d_{y}\right]}^{T}$, respectively. In addition, the loading force and the corresponding normal force to the moment lever are represented by $u_{1}$ and $u_{2}$, respectively. Note that for the logarithmic deformation, an adequate profile of the activator surface should be designed to satisfy kinematic and static constraints. As the contact point is coincident regardless of coordinate system, it has the following relationship:

(6) $$R_{1}^{0}p^{1}=d^{0}+R_{2}^{0}p^{2}$$

where $p^{i}\left(={\left[\begin{array}{cc} x_{c}^{i} & y_{c}^{i} \end{array}\right]}^{T}\right)$ is the contact vector with respect to the $\Sigma_{i}$. $R_{j}^{i}$ is the rotation matrix from the *i*-th coordinate to the *j*-th coordinate. $d^{0}\left(={\left[\begin{array}{cc} d_{x}^{0} & d_{y}^{0} \end{array}\right]}^{T}={\left[\begin{array}{cc} l_{1}+l_{2} & h \end{array}\right]}^{T}\right)$ denotes the origin of the measurement unit with respect to the $\Sigma_{0}$.

The contact surface should be tangential to the moment lever and equal to the slope of the moment lever as follows:

(7) $${\frac{sin\theta_{1}+cos\theta_{1}f^{\prime }\left(x\right)}{cos\theta_{1}-sin\theta_{1}f^{\prime }\left(x\right)}|}_{x=x_{c}^{1}}=\frac{d_{y}^{0}-y_{c}^{0}}{d_{x}^{0}-x_{c}^{0}}=-tan\theta_{2}$$

where f(x) is the curvature of the activator with respect to the $\Sigma_{1}$. When the forces are in equilibrium and the friction force is negligible, the resultant moment of the activator becomes zero.

(8) $$\left(R_{1}^{0}\left[\begin{array}{c} l\\ 0\\ 0 \end{array}\right]\right)\times \left[\begin{array}{c} 0\\ u_{1}\\ 0 \end{array}\right]-\left(R_{1}^{0}p^{1}\right)\times \left(R_{2}^{0}\left[\begin{array}{c} 0\\ u_{2}\\ 0 \end{array}\right]\right)=0$$

Also from Hook’s law, the torque is proportional to the angular displacement, as follows:

(9) $$-u_{2}x_{2}=k_{s}\theta_{2}$$

where $k_{s}$ is the stiffness coefficient. (6) and (7) imply the kinematic constraints, while (8) and (9) mean the static constraints. By solving these equations for the particular profile f(x), the corresponding strain curve is determined. To obtain the optimal curved profile whose strain curve is best fit to the logarithmic curve, the profile is designed by an exponential function as follows:

(10) $$p_{k}^{1}={\left[x_{k}^{1}\;\;\;y_{k}^{1}\right]}^{T}$$

with

$$x_{k}^{1}=l_{0}+k\Delta x,\;\;\;y_{k}^{1}=\left(1-a^{x_{k}^{1}-x_{N}^{1}}\right)h$$

where *k* (= 0, 1, 2, …, *N*) is the index of the sample for the profile. $p_{k}^{1}$ is the sampled position on the activator profile to compare with the reference logarithmic curve. $l_{0}$ is the initial point and Δx is the interval between two consecutive sampled data points, $x_{k}^{1}$. a is the base of the exponential function.

### 2.3. Optimization of Design Parameters

By an evolutionary optimization, the design parameters of the proposed load cell model, $l_{0}$, $l_{1}$, $l_{2}$, h, and a are determined to minimize the following objective function:

(11) $$g=w_{0}\overset{N}{\underset{k=1}{\sum }}\frac{\left|\theta_{ref}^{k}-\theta_{2}^{k}\right|}{N}+w_{1}\theta_{1}^{N}+w_{2}\theta_{2}^{N}$$

where $w_{0}$, $w_{1}$, and $w_{2}$ are weight factors. $\theta_{ref}^{k}$ is the desired angle of the torque measurement unit according to the loading force $u_{1}$, which indicates the reference strain curve. $\theta_{1}^{N}$ and $\theta_{2}^{N}$ denote the angles of the activator and the torque measurement unit, respectively, at *k* = *N*. For the evolutionary optimization, the weight factors, $w_{0}$, $w_{1}$, and $w_{2}$ were set as 100.0, 20.0, and 4.0, respectively, to make the scales of the terms in the objective function comparable. The maximum number of generations and the population size were set as 100 and 30, respectively. The stiffness coefficient $k_{s}$ was set as 2915.5 mNm/rad.

**Figure 2:**
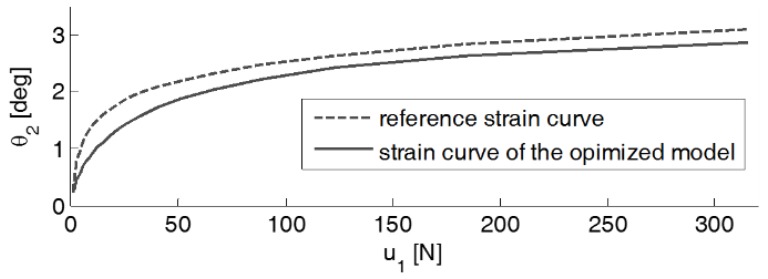
Comparison between the reference and optimized strain curves.

As a result of the evolutionary optimization, the optimized values for the parameters, $l_{0}$, $l_{1}$, $l_{2}$, h, and a, were obtained as 20.89 mm, 34.90 mm, 0.90 mm, 0.50 mm, and 1.14, respectively. Figure 2 shows the variation of the angle of the torque measurement unit, $\theta_{2}$, according to the loading force $u_{1}$. This is interpreted as the strain curve of the optimized model. From the figure, it can be shown that the optimized strain curve is similar to the reference one, which means that the proposed load cell adequately approximates the logarithmic deformation of the loading force. In addition, the angular displacements of the mechanical parts in motion is significantly small; therefore, this optimized model is practical enough for high stroke systems.

## 3. FEM Analysis

**Figure 3:**
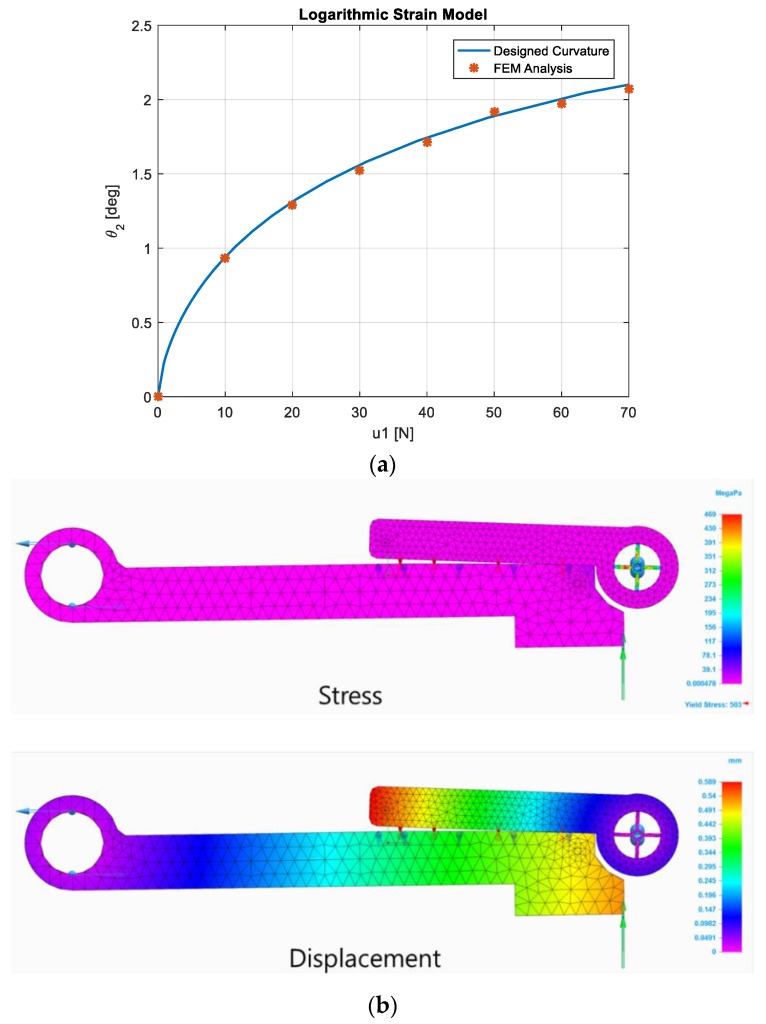
FEM Analysis. (**a**) Strain curvature. (**b**) FEM snapshot ($u_{1}=60\;N$).

In order to verify the feasibility and performance of the proposed strain model, FEM analysis was implemented. The same curved profile, shape of the activator and deformation part, $l_{0}$, $l_{1}$, $l_{2}$, h, and a, were applied, as derived by the simulation result. The material for the sensor frame was aluminum alloy (Al7075) and modulus of elasticity and yield stress were 71,705.473 MPa and 503.317 MPa, respectively. Mesh type and size were chosen as tetrahedral mesh and 0.5 mm, respectively. Activator center, $\Sigma_{1}$, was constrained as free rotation for only the hinge direction, with no degree of freedom for the other rotation and translation, while the center of the deformation part was totally fixed. The contact surface was set to prevent inter-penetration. Loading force was applied from 0 to 70 N, increasing by 10 N. Figure 3a shows the strain curve of the deformation part. The solid line and asterisk indicate a designed curve and FEM result, respectively. Figure 3b shows a stress and deformation example. The FEM result was very similar to the designed one with an admissible small difference, which was caused by mesh approximation and sensor frame, which was assumed to be rigid, with an elastic body.

## 4. Conclusions

This paper proposed a nonlinear load cell that deforms along a logarithmic strain curve with respect to an applied force. The load cell was composed of the activator and the linear torque measurement unit. The activator receives the loading force and makes consequent rotational motions, keeping contact with the moment lever. As the exerted force increases, the contact point moves toward the center of the lever arm of the torque measurement unit. Consequently, the moment arm becomes shortened as the loading force is continually applied. This enables a change in the sensitivity of load cell throughout the force measurement range. A mathematical formulation of the proposed load cell was derived to describe the relationship between the loading force and the consequent strain of the torque measurement unit. To fit the strain behavior to the logarithmic curve, the profile of the activator surface was designed as an exponential function and the optimal design parameters were obtained by the evolutionary computation.

In this paper, the mechanical structure and design method for the nonlinear load cell were proposed. Manufacturing the sensor and its experimental analysis are future works.

## References

[1] Kim N., Lee B. (2019). Stack structured hybrid load cell for a high-stroke system. J. Adv. Mech. Des. Syst. Manuf..

[2] Muller I., de Brito R.M., Pereira C.E., Brusamarello V. (2009). Load cells in force sensing analysis-theory and a novel application. IEEE Instrum. Meas. Mag..

[3] Demetropoulos C.K., Morgan C.R., Sengupta D.K., Herkowitz H.N. (2009). Development of a 4-axis load cell used for lumbar interbody load measurements. Med. Eng. Phys..

[4] Adami A.M., Pavel M., Hayes T.L., Singer C. (2009). Detection of movement in bed using unobtrusive load cell sensors. IEEE Trans. Inf. Technol. Biomed..

[5] Lee W., Yoon H., Han C., Joo K.M., Park K.S. (2016). Physiological signal monitoring bed for infants based on load-cell sensors. Sensors.

[6] Balbinot A., Milani C., Nascimento J. (2014). A new crank arm-based load cell for the 3D analysis of the force applied by a cyclist. Sensors.

[7] Lee Y.-F., Chu C.-Y., Xu J.-Y., Lan C.-C. (2016). A humanoid robotic wrist with two-dimensional series elastic actuation for accurate force/torque interaction. IEEE/ASME Trans. Mechatron..

[8] Schmitz A., Maggiali M., Natale L., Bonino B., Metta G. A tactile sensor for the fingertips of the humanoid robot iCub. Proceedings of the IEEE/RSJ International Conference on Intelligent Robots and Systems.

[9] Park H., Lee B., Kim D. (2016). Development of Anthropomorphic Robot Finger for Violin Fingering. ETRI J..

[10] Park I.-W., Kim J.-Y., Lee J., Oh J.-H. (2007). Mechanical design of the humanoid robot platform, HUBO. Adv. Robot..

[11] Englsberger J., Werner A., Ott C., Henze B., Roa M.A., Garofalo G., Burger R., Beyer A., Eiberger O., Schmid K. Overview of the torque-controlled humanoid robot TORO. Proceedings of the 2014 IEEE-RAS International Conference on Humanoid Robots.

[12] Banala S.K., Kim S.H., Agrawal S.K., Scholz J.P. Robot assisted gait training with active leg exoskeleton (ALEX). Proceedings of the 2nd IEEE RAS & EMBS International Conference on Biomedical Robotics and Biomechatronics.

[13] Mastinu G., Gobbi M., Previati G. (2011). A new six-axis load cell. Part I: design. Exp. Mech..

[14] Gobbi M., Previati G., Guarneri P., Mastinu G. (2011). A new six-axis load cell. Part II: Error analysis, construction and experimental assessment of performances. Exp. Mech..

[15] Ballo F., Gobbi M., Previati G., Mastinu G. (2014). Advances in force and moments measurements by an innovative six-axis load cell. Exp. Mech..

